# Advancing omics technologies in acute respiratory distress syndrome: paving the way for personalized medicine

**DOI:** 10.1186/s40635-025-00766-4

**Published:** 2025-06-13

**Authors:** Lou’i Al-Husinat, Mohammad Araydah, Sarah Al Sharie, Saif Azzam, Denise Battaglini, Arqam Alrababah, Rana Haddad, Khaled Al-Asad, Claudia C. Dos Santos, Marcus J. Schultz, Fernanda F. Cruz, Pedro L. Silva, Patricia R. M. Rocco

**Affiliations:** 1 Department of Clinical Sciences, Faculty of Medicine, Yarmouk University, Irbid, Jordan; 2 Department of Internal Medicine, Istishari Hospital, Amman, Jordan; 3 Office of Scientific Affairs and Research, King Hussein Cancer Center, Amman, Jordan; 4 Faculty of Medicine, Yarmouk University, Irbid, Jordan; 5 Department of Surgical Sciences and Integrated Diagnostics (DISC), University of Genova, Genoa, Italy; 6 Anesthesia and Intensive Care, IRCCS Ospedale Policlinico San Martino, Genoa, Italy; 7 Department of General Surgery, Abdali Hospital, Amman, Jordan; 8 Faculty of Medicine, Jordan University of Science and Technology, Irbid, Jordan; 9 The Keenan Research Centre for Biomedical Science of St. Michael’s Hospital, Toronto, ON Canada; 10 Institute of Medical Sciences and Interdepartmental Division of Critical Care, Faculty of Medicine, University of Toronto, Toronto, ON Canada; 11 Department of Intensive Care, Amsterdam UMC, Locatie AMC, Amsterdam, The Netherlands; 12 Department of Anesthesia, General Intensive Care and Pain Management, Medical University Wien, Vienna, Austria; 13 Nuffield Department of Medicine, University of Oxford, Oxford, UK; 14 Mahidol-Oxford Research Unit (MORU), Mahidol University, Bangkok, Thailand; 15 Laboratory of Pulmonary Investigation, Carlos Chagas Filho Institute of Biophysics, Federal University of Rio de Janeiro, Rio de Janeiro, Brazil

**Keywords:** Acute respiratory distress syndrome, Genomics, Transcriptomics, Proteomics, Metabolomics, Lipidomics, Personalized medicine

## Abstract

Despite advances in critical care, acute respiratory distress syndrome (ARDS) remains a potentially life-threatening condition with high mortality. The heterogeneous nature of ARDS, caused by diverse etiologies, poses considerable challenges to accurate diagnosis, treatment, and prognosis. Conventional methods often fail to elucidate the pathophysiology of ARDS, thus limiting therapeutic efficacy. However, recent advances in omics technologies, including genomics, transcriptomics, proteomics, metabolomics, lipidomics, and epigenomics, have provided deeper insights into ARDS mechanisms. Genomic studies have identified genetic variants associated with ARDS susceptibility, such as polymorphisms in genes encoding angiotensin-converting enzyme, surfactant proteins, toll-like receptor 4, interleukin-6, Fas/FasL, and vascular endothelial growth factor, offering potential therapeutic targets. Transcriptomic and proteomic reveal distinct biomarker profiles associated with ARDS pathogenesis, including dysregulated inflammatory signaling, epithelial and endothelial barrier dysfunction, and compromised immune responses. Metabolomics has highlighted biomarkers, such as phenylalanine and choline, aiding in severity assessment, subphenotype stratification, and treatment response prediction. Lipidomics has uncovered disruptions in lipid metabolism, including altered phospholipids, sphingolipids, and eicosanoids, with key lipid species such as lysophosphatidylcholine and ceramide emerging as biomarkers for severity and outcomes. Epigenomics explores DNA methylation, histone modifications, and non-coding RNAs, revealing their role in regulating inflammation, immune responses, and tissue repair in ARDS. These epigenetic changes hold promise for biomarker discovery and personalized therapy. Integrating these omics technologies advances our understanding of ARDS pathophysiology, enabling precision medicine approaches. This review examines the latest advancements in omics research related to ARDS, emphasizing its role in developing personalized diagnostics and therapeutic strategies to improve disease monitoring, prognosis, and treatment outcomes.

**Supplementary Information:**

The online version contains supplementary material available at 10.1186/s40635-025-00766-4.

## Introduction

Acute respiratory distress syndrome (ARDS) is a complex complication of a critical illness, characterized by increased alveolar–capillary permeability, lung edema, yielding respiratory failure [1]. It represents a significant challenge in critical care medicine, arising from pulmonary insults, such as pneumonia or aspiration, and extrapulmonary causes, including sepsis or trauma [2]. Despite extensive research, ARDS remains associated with high mortality rates, exceeding 40% in severe cases [3].

The recently introduced ‘New Global Definition of ARDS’ [1] incorporates simplified criteria, including peripheral oxygen saturation to fraction of inspired oxygen (SpO₂/FiO₂) ratio and lung ultrasound, making diagnosis feasible even in resource-limited environments. However, this definition does not address the biological heterogeneity of ARDS, which significantly influences patient outcomes and highlights the need for personalized approaches.

Advances in precision medicine, particularly the growing accessibility of "omics" technologies, have opened new avenues for understanding the pathophysiology of ARDS [4]. Omics approaches, including genomics, transcriptomics, proteomics, metabolomics, lipidomics, and epigenomics, enable comprehensive analysis of the disease's complexity, identifying biomarkers, elucidating critical pathways, and suggesting novel therapeutic targets [5].

Omics technologies have advanced the understanding of ARDS by identifying key molecular alterations. Proteomics and metabolomics reveal dysregulated proteins and metabolic pathways linked to inflammation and disease progression. Transcriptomics, genomics, and lipidomics highlight genetic variants, gene expression patterns, and lipid biomarkers associated with ARDS severity and outcomes. Epigenomics further uncovers regulatory mechanisms influencing immune responses and tissue repair [6].

This review examines the latest advancements in omics research in ARDS, highlighting how these methodologies enhance our understanding of its molecular mechanisms. It also underscores their role in advancing precise diagnostics, prognostics, and personalized therapeutic strategies.

### Genomics

Genetic studies investigate genes and inherited variations, such as single nucleotide polymorphisms (SNPs), which may significantly alter protein structure and function, contributing to specific phenotypes. Approaches such as candidate gene studies, genome-wide association studies (GWAS), whole-genome sequencing (WGS), and whole-exome sequencing (WES) have identified genetic variants linked to ARDS risk, severity and outcomes [7].

Candidate gene studies explore specific genetic variations associated with phenotypic traits [8]. While often limited by small sample sizes and lack of reproducibility, they have contributed to valuable insights [9]. Over the past year, several candidate gene studies identified variants influencing ARDS risk and mortality (Supplementary Table 1). In this line, the single-nucleotide polymorphism (SNP) rs9984273 in the interferon alpha/beta receptor (IFNAR) has been associated with coronavirus disease 2019 (COVID-19)-related ARDS [10]. The minor allele was correlated with higher IFNAR expression, faster declines in interferon-gamma and interleukin (IL)-6 levels, and improved survival in ARDS patients treated with interferon-beta. However, the restricted scope of candidate gene studies limits their broader applicability [9].

Genome-wide association studies have identified multiple genes associated with risk and severity of ARDS (Supplementary Table 2), including the protein tyrosine phosphatase receptor type F polypeptide (*PTPRF*), interacting protein 1 (*PPF1A1*), selectin P ligand (*SELPL*), FMS-related receptor tyrosine kinase 1 (*FLT1*), BLOC-1-related complex subunit 5 (*BORCS5*), dual specificity phosphatase 16 (*DUSP16*), and Fps/Fes-related (*FFR*) genes. One of the first GWAS studies in ARDS analyzed 620,901 SNPs in trauma patients and identified SNP rs471931 in PPFIA1 as being associated with ARDS [11]. This association was validated in a smaller replication cohort, suggesting a functional role in ARDS susceptibility. However, the use of pediatric controls in the discovery phase and modest sample sizes posed limitations. A recent trans-ancestry GWAS meta-analysis in COVID-19 patients identified eight genes (VWA8, PDE8B, CTSC, THSD7B, STK39, FBXO34, RPL6P27, METTL21C) significantly associated with hospitalization, further demonstrating the utility of GWAS in recognizing genetic markers.

Whole-genome sequencing and whole-exome sequencing offer more detailed insights into genetic variants. A WES case–control study compared 1.38 million SNPs between 96 ARDS patients and 1000 healthy controls, identifying variants, such as rs78142040 in the ARDS gene and rs9605146 in the XKR3 gene, associated with increased ARDS susceptibility and mortality. However, low variant frequencies in control populations, small sample sizes, and population differences limited this study's generalizability [12].

Another WES study analyzed genetic polymorphisms in 105 ARDS patients, linking SNPs in genes, such as AKR1B10, PIEZO2, and MYL3 to ARDS outcomes. Pathways implicated included ECM–receptor interaction, purine metabolism, platelet activation, and adrenergic signaling [7]. These findings highlight WES's potential for identifying genetic mechanisms underlying ARDS.

Polygenic Risk Scores (PRS) are a quantitative metric that estimates an individual's genetic predisposition to a particular trait or disease. They are derived by aggregating the effects of multiple genetic variants, usually single nucleotide polymorphisms, across the genome. PRSs estimate an individual’s genetic predisposition to disease by integrating the effects of multiple genetic variants [13]. PRSs facilitate early monitoring, preventive interventions, and personalized treatments based on genetic risk profiles.

In summary, genetic studies have provided critical insights into ARDS pathogenesis, identifying key genes and pathways linked to disease risk and progression. However, challenges such as small sample sizes, high costs, and the need for large, diverse data sets hinder broader application. Despite these limitations, genomics holds transformative potential for advancing personalized care in ARDS, enabling tailored interventions based on individual genetic profiles.

### Transcriptomics

Transcriptomics, the study of ribonucleic acid (RNA) transcripts produced by the genome under specific conditions, offers a dynamic view of gene expression compared to the static insights of genomics [14]. This field includes both messenger RNA (mRNA) and non-coding RNAs, such as long non-coding RNAs (lncRNAs) and microRNAs (miRNAs), reflecting advancements in RNA biology [8]. Unlike genomics, transcriptomics captures real-time RNA fluctuations in response to internal and external stimuli, enabling detailed insights into disease mechanisms across tissues and even at the single-cell level [15].

In patients with ARDS, transcriptomics has revealed altered levels of mRNAs, such as MyD88, IRAK1, NF-κB, IL-6, CASP1, IL18, and p300/CBP. A study of 104 ARDS patients highlighted alternative splicing in toll-like receptor (TLR) signaling genes, with a shift favoring the pro-inflammatory MyD88L isoform over the anti-inflammatory MyD88S isoform, and higher levels of the anti-inflammatory IRAK1c associated with improved survival [16]. In addition, elevated mRNAs for p300 and CBP were linked to IL-17-driven inflammation and predicted higher mortality, emphasizing their role in ARDS pathophysiology [17]. Transcriptomic analyses have also identified distinct gene expression profiles between ARDS and sepsis, with genes such as NKG7 and IFI27 emerging as potential biomarkers for differentiation [18].

A prospective observational study of 34 ARDS patients and 15 healthy controls identified significant transcriptomic dysregulation, with 1,233 mRNAs and 6 miRNAs upregulated, and 1,580 mRNAs and 13 miRNAs downregulated. Analysis revealed alterations in immune-related pathways, including upregulation of the programmed cell death ligand 1 (PD-L1) and programmed death-1 (PD-1) cancer immunotherapy pathways and suppression of the Th1 pathway, indicating an imbalanced immune response in ARDS [19].

#### The role of MicroRNAs in ARDS pathophysiology and therapeutic potential

MicroRNAs (miRNAs), a class of non-coding RNAs that regulate gene expression, are key players in ARDS pathophysiology, influencing processes, such as inflammation, cell death, and immune responses [20]. Clinical studies have identified miRNAs with prognostic and diagnostic value in ARDS (Supplementary Table 3). In this line, elevated miR-181a and miR-92a levels were associated with increased ARDS risk, while elevated miR-424 levels correlated with reduced risk [21]. Other studies linked decreased levels of miR-141-3p to worse outcomes, including pulmonary fibrosis and impaired lung function [22].

Despite promising findings, miRNA-based therapies for ARDS remain restricted to pre-clinical settings [23]. Certain miRNAs have been found to either worsen or alleviate ARDS pathology, making them promising targets for therapeutic intervention [24]. Strategies to modulate these miRNAs involve using delivery systems like lipid nanoparticles (LNPs), which transport miRNA mimics or inhibitors directly to the lungs [24]. LNPs protect miRNAs from degradation and improve their cellular uptake, thereby increasing therapeutic effectiveness. The successful use of LNPs in mRNA vaccines, such as those for SARS-CoV-2, highlights their potential for advancing miRNA-based treatments for ARDS [24].

Similarly, extracellular vesicles (EVs) derived from mesenchymal stromal cells (MSCs) carry miRNAs, such as miR-21-5p, miR-27a-3p, and miR-181a-5p, mediating anti-inflammatory and tissue repair effects in ARDS models [23].

Despite these advancements, transcriptomics and miRNA research face challenges. These include the complexity of data analysis, patient heterogeneity, timing of sample collection, and accessibility to high-quality tissue samples from critically ill patients. Transcriptomic studies also capture only a single timepoint snapshot, missing dynamic changes over the disease course [25].

Looking forward, miRNA-based interventions and transcriptomic insights hold great potential for developing targeted diagnostic and therapeutic strategies for ARDS. Integrating advanced delivery systems, such as LNPs and MSC-derived EVs, with robust bioinformatics tools may pave the way for translating these findings into clinical practice.

#### Divergent transcriptional profiles of lung and blood monocytes

To better understand the immune response in ARDS, one study investigated the transcriptional differences between alveolar macrophages (AMs) and peripheral blood monocytes (PBMs), aiming to determine whether circulating immune cells accurately reflect lung-specific immune activity [26]. Using genome-wide expression analysis, the researchers identified over 6,000 differentially expressed genes, revealing distinct transcriptional profiles between AMs and PBMs [26].

Notably, AMs from patients with better clinical outcomes were enriched in proinflammatory and immune activation pathways, whereas PBMs had a significantly lower immune signature [26]. On the other hand, PBMs from patients with worse clinical outcomes demonstrated elevated inflammatory signatures, suggesting a correlation between systemic inflammation and disease severity [26]. These findings highlight the limitations of relying on blood-based transcriptomic analyses in ARDS and emphasize the need for lung-targeted molecular profiling to improve phenotyping, biomarker discovery, and precision medicine approaches [26].

### Proteomics

Proteomics involves the large-scale study of proteins and their expression patterns across biological systems, offering critical insights into the molecular mechanisms underlying diseases, such as ARDS [27]. This approach has proven helpful in identifying biomarkers and therapeutic targets through the analysis of blood, exhaled breath, lung tissue, and bronchoalveolar lavage fluid (BALF) [27]. A key advantage of proteomics over other omic fields is its direct analysis of biologically active molecules, which are transcribed from mRNA and play functional roles in disease processes [27]. Since proteins drive cellular responses and disease progression, proteomics holds significant potential for identifying early biomarkers and treatment targets in ARDS, making it an invaluable tool for enhancing patient stratification and precision medicine approaches [27].

Proteomic research in ARDS can be targeted or untargeted, depending on the research objectives [28]. Untargeted proteomics (discovery proteomics) is useful for identifying a wide range of proteins linked to disease progression, detecting potential biomarkers, and enabling large-scale studies [28]. However, its ability to accurately quantify protein levels is limited. In contrast, targeted proteomics focuses on specific, preselected proteins, providing greater sensitivity and precision in measuring their levels across different ARDS subphenotypes [28]. While targeted proteomics offers more accurate quantification, it may overlook novel biomarkers due to its narrower focus [28].

The first proteomic analysis in ARDS revealed the overexpression of various proteins in plasma, BALF, and edema fluid of ARDS patients [29]. Proteomics could distinguish ARDS subphenotypes, such as direct vs. indirect lung injury, by identifying unique protein expression profiles [29]. An exploratory study of serum-derived extracellular vesicles in patients with COVID-19 identified potential markers of disease severity, including Von Willebrand factor, serum amyloid A-2, histone H4, H2A type 2-C, and fibrinogen β-chain [30].

Proteomic analysis of BALF from ARDS patients has revealed significant variations in protein expression associated with lung injury and disease progression [31]. Proteins involved in coagulation, fibrinolysis, immune responses, and inflammation were upregulated in ARDS, with differences observed between survivors and non-survivors.

In short, proteomics plays a vital role in identifying biomarkers and therapeutic targets for ARDS by uncovering distinct protein profiles across subphenotypes and disease severities. Complementary advancements in proteomics and peptidomics have highlighted key proteins and peptides involved in inflammation, coagulation, and immune responses, offering valuable insights into disease progression and prognosis. These tools hold immense potential to advance personalized medicine by enabling the development of targeted diagnostic and therapeutic strategies for ARDS [32].

### Metabolomics

Metabolomics, the comprehensive analysis of metabolites in biological specimens, is an emerging technology that holds significant potential in advancing precision medicine for complex conditions like ARDS. By identifying metabolic changes linked to disease mechanisms, metabolomics can aid in diagnosing ARDS, assessing disease severity, and evaluating treatment responses [33]. Key analytical techniques include nuclear magnetic resonance (NMR) spectroscopy, gas chromatography–mass spectrometry (GC–MS), and liquid chromatography–mass spectrometry (LC–MS) [34]. In ARDS research, metabolomics is applied across various samples—including exhaled breath, lung tissue, blood, BALF, and pleural effusion fluid—each providing distinct insights into the disease pathophysiology [35].

Metabolomic analysis can be categorized into two approaches: targeted and untargeted. Targeted metabolomics focuses on specific metabolites within known pathways, whereas untargeted metabolomics explores metabolites without a predefined hypothesis, offering a more exploratory perspective [36].

Early studies in metabolomics demonstrated its potential in ARDS research. In exhaled breath of ARDS patients, Bos et al. employed untargeted GC–MS to analyze 500 metabolites, identifying elevated levels of 3-methylheptane, octane, and acetaldehyde, indicative of oxidative stress, though these findings did not differentiate between direct and indirect ARDS or correlate with disease severity [37].

In sepsis-induced mild ARDS, 1H-NMR analysis revealed increased plasma levels of glutathione, adenosine, and phosphatidylserine, alongside decreased sphingomyelin, reflecting oxidative stress and endothelial injury [38]. A GC–MS study of 222 metabolites in 37 ARDS patients further revealed that phenylalanine, aspartic acid, and carbamic acid levels correlated with disease severity [39].

Recent advances have expanded the scope of metabolomics in ARDS. Ultrahigh-performance liquid chromatography–tandem mass spectrometry (UHPLC–MS/MS) has been used to profile blood metabolites in ARDS patients, revealing distinct metabolic profiles that differentiate survivors from non-survivors. Non-survivors showed significantly elevated levels of phenylacetylglutamine, D-phenylalanine, and phenylalanine, shedding light on metabolic alterations linked to poor outcomes [40]. Specific metabolites such as choline, 3-hydroxybutyrate, citrate, acetoacetate, acetone in serum, and 1-methylnicotinamide in urine have been identified as predictors of treatment responses in ARDS caused by community-acquired pneumonia [41]. In addition, plasma metabolic profiling in ARDS patients with different infectious etiologies, including H1N1 influenza, COVID-19, and bacterial pneumonia, has demonstrated variations in pathways, such as taurine and hypotaurine metabolism, tricarboxylic acid cycle intermediates, and glycerophospholipids, highlighting metabolomics’ potential to distinguish ARDS etiology and severity.

Metabolomics has also been applied to BALF and edema fluid analysis. Using 1H-NMR, researchers identified significant differences in ethanol, alanine, and choline levels in ARDS patients [42]. LC–MS studies revealed elevated levels of lactic acid, guanosine, and phosphatidylcholine in BALF, consistent with inflammation and oxidative stress [43]. However, edema fluid studies using UHPLC–MS/MS found no significant differences between ARDS patients and controls with hydrostatic pulmonary edema. Instead, hypermetabolic and hypometabolic subtypes were identified, with hypermetabolic profiles associated with non-pulmonary sepsis [44].

NMR spectroscopy has been employed to quantify metabolites, such as proline, taurine, and glutamate in BALF, distinguishing mild ARDS from moderate/severe cases, despite some overlap in metabolite levels [45]. Further research has demonstrated the potential of metabolomics to differentiate ARDS severity and pulmonary vs. extrapulmonary subtypes using serum and BALF metabolite profiles, though analytical challenges remain [46].

Despite its promise, metabolomics faces several challenges in ARDS research [47]: (1) data complexity: the high dimensionality of metabolomic data and multicollinearity among metabolites complicate analysis and interpretation; (2) variability across studies: inconsistencies across studies, due to small sample sizes, lack of external validation, and methodological differences, limit reproducibility; and (3) sample collection and practicality: certain sample types, such as BALF, may be less feasible for routine use in critically ill patients, and metabolomic profiles can vary based on sample source and disease stage [48].

In short, metabolomics has already significantly advanced our understanding of ARDS by identifying potential biomarkers, elucidating injury-specific profiles, and offering insights into real-time disease processes. As methodologies improve and limitations are addressed, metabolomics holds great promise for enhancing ARDS diagnosis, stratification, and treatment strategies.

### Lipidomics

Lipidomics is a branch of omics sciences dedicated to the comprehensive analysis of lipids in biological systems, including their identification, quantification, and functional roles in cellular processes [49]. Lipid metabolism is important for maintaining cell membrane integrity, supporting cellular signaling pathways, and regulating energy homeostasis. Disruptions in lipid metabolism, including alterations in phospholipids, sphingolipids, and eicosanoids, significantly contribute to the pathogenesis and progression of ARDS [50].

Phospholipid imbalances in ARDS, particularly altered levels of phosphatidylcholine and lysophosphatidylcholine, increase membrane permeability and worsen lung injury [51]. In addition, disruptions in sphingolipids, such as ceramide accumulation, amplify inflammation and cell death, while excessive pro-inflammatory eicosanoids, including prostaglandins and leukotrienes, drive the inflammatory cascade and lung damage [52].

Certain lipid species, including lysophosphatidylcholine, ceramide, and oxidized phospholipids, have been proposed as biomarkers for assessing ARDS severity and predicting outcomes [53]. Elevated plasma and BALF levels of lysophosphatidylcholine correlate with poor outcomes in ARDS, while higher ceramide levels are associated with increased mortality and may serve as prognostic markers for disease severity [54]. Oxidized phospholipids, markers of oxidative stress, have also been linked to alveolar epithelial damage in ARDS [55].

A study examined lipid profile changes in plasma and erythrocytes from patients with sepsis and septic shock, finding significant alterations when compared to healthy controls [56]. Specifically, lysophosphatidylcholines and sphingomyelins were downregulated, while both saturated and unsaturated phosphatidylcholines increased in plasma and erythrocytes of septic patients [57]. These lipidomic shifts suggest that lysophosphatidylcholines and sphingomyelins play a role in sepsis pathogenesis, and their altered levels could serve as sensitive biomarkers [58].

In short, lipidomics offers valuable insights into lipid dysregulation in ARDS and has already revealed potential biomarkers of disease severity, such as lysophosphatidylcholine and ceramide. However, the variability in lipid profiles across studies and technical challenges in lipid identification and quantification complicate data interpretation. In addition, inconsistencies between studies may limit the applicability of these findings.

### Epigenomics

Epigenomics investigates changes in gene activity or expression that do not involve alterations to the underlying DNA sequence, but instead occur through mechanisms, such as DNA methylation, histone modification, and non-coding RNA regulation [59]. These changes can be inherited or influenced by environmental factors, without altering the genetic code itself [60].

One of the most studied epigenetic modifications is DNA methylation, particularly at cytosine–phosphate–guanine (CpG) sites [61]. DNA methylation regulates gene expression, including genes involved in inflammation, immune responses, and tissue repair [61]. In ARDS, altered DNA methylation, including hypermethylation of anti-inflammatory genes and hypomethylation of pro-inflammatory cytokines, disrupts immune regulation and exacerbates lung injury [6]. A multi-microarray analysis conducted by Zhang et al. identified 44,439 DNA methylation alterations and 29 differentially expressed mRNAs in ARDS patients, uncovering key epigenetic modifications that impact inflammation, endothelial function, and coagulation regulation [62]. Several of these methylation sites exhibited high diagnostic efficacy, with AUC values reaching up to 0.99, positioning them as promising biomarkers for differentiating ARDS patients from healthy individuals. These findings suggest that DNA methylation patterns could serve as potential biomarkers for both the diagnosis and prognosis of ARDS.

Histone modifications such as acetylation, methylation, and phosphorylation influence chromatin structure and gene expression by altering DNA accessibility [63]. In ARDS, changes in histone modifications affect genes involved in inflammation and epithelial–mesenchymal transition (EMT), a key process in lung fibrosis [64].

In addition, non-coding RNAs like microRNAs regulate gene expression, affecting inflammation, apoptosis, and cellular repair [65]. Although the role of non-coding RNAs in ARDS is still under investigation, they hold potential as biomarkers or therapeutic targets [66].

In short, epigenomic biomarkers could significantly enhance the diagnosis, prognosis, and treatment of ARDS. Identifying DNA methylation patterns could offer early indicators of ARDS susceptibility or severity. While epigenomic research faces challenges in sampling and analysis, its potential to contribute novel insights and therapeutic targets for personalized management of ARDS is immense.

### Subphenotyping in ARDS and the application of omics

Acute Respiratory Distress Syndrome (ARDS) is a highly heterogeneous condition, characterized by diverse etiologies, clinical presentations, and varying responses to treatment. This variability presents substantial challenges in identifying effective therapeutic strategies. Subphenotyping ARDS, however, holds promise in stratifying patients into distinct clinical, physiological, or biological subgroups, thereby facilitating more personalized approaches to treatment [67–69]. Clinical subphenotyping in ARDS categorizes patients based on common etiological factors, disease progression, or radiographic characteristics [69]. Physiologic subphenotyping classifies patients according to the severity of lung impairment [69]. Biological subphenotyping refers to the classification of patients based on shared host response patterns, aiming to identify molecularly distinct subgroups with similar pathophysiological mechanisms [69].

Various biomarkers, including genomic, transcriptomic, and metabolic markers, have been explored to subphenotype ARDS. However, protein biomarkers are most commonly used, as they provide the primary basis for distinguishing biologically distinct subphenotypes. For example, a latent class analysis (LCA) of clinical and protein biomarker data from the ARMA [70] and ALVEOLI [71] trials identified two distinct biological ARDS subphenotypes, termed “hyperinflammatory” and “hypoinflammatory”, each associated with different clinical outcomes [72]. Further analyses of data from five major ARDS trials—ARMA, ALVEOLI, FACTT, SAILS, and HARP-2—along with two prospective observational cohorts, including over 4,000 patients, consistently showed that patients with the hyperinflammatory subphenotype had higher mortality rates compared to those with the hypoinflammatory subphenotype [73–75]. Beyond its prognostic significance, these subphenotypes also exhibited differential responses to therapies, such as PEEP, fluid management strategies, and simvastatin, as shown in secondary analyses of completed trials [73–75].

In addition, an independent study using cluster analysis of protein biomarker data from a large Dutch ICU cohort identified two molecular subphenotypes: “reactive” and “uninflamed” [76]. The reactive subphenotype, characterized by elevated levels of IL-6, Ang-1 and 2, PAI-1, and interferon-gamma, was associated with worse clinical outcomes compared to the uninflamed subphenotype [76].

Despite the progress in ARDS subphenotyping based on systemic biomarkers, it remains unclear whether these classifications truly reflect immune responses within the lung [77]. One study investigated whether systemic ARDS subphenotypes, previously classified as hyperinflammatory vs. hypoinflammatory (LCA model) or reactive vs. uninflamed (cluster model), correspond to differences in alveolar inflammation [77]. Surprisingly, the researchers found no significant differences in key inflammatory mediators (IL-6, IL-8, TNF-α, and IFN-γ) within the alveolar compartment between subphenotypes, even though overall cytokine levels in lung fluid were higher than in plasma [77]. In addition, lung microbiome composition did not differ significantly between subphenotypes, although a trend indicated a higher prevalence of Enterobacteriaceae in the hyperinflammatory group [77]. These results challenge the assumption that systemic subphenotypes fully reflect lung-specific immune responses, highlighting the need for pulmonary-targeted phenotyping to improve ARDS classification and guide precision medicine approaches.

Transcriptomics has also been employed to phenotype ARDS, with studies identifying distinct gene expression patterns that differentiate molecular subgroups. A study by Bos et al. analyzed leukocyte gene expression profiles and identified two distinct ARDS subphenotypes based on immune responses: one with profound inflammatory activation and another with a more muted immune response [78]. The hyperinflammatory phenotype was associated with higher expression of genes related to innate immunity, cytokine signaling, and neutrophil activation, and was linked to worse clinical outcomes, including higher mortality [78].

Yeyha et al. applied K-means clustering to whole blood microarray data from 96 pediatric ARDS patients, identifying three distinct subphenotypes, termed Critical Illness-Associated Transcriptomic Subgroups (CATS 1–3) [79]. CATS 1 showed enrichment in adaptive immune and T-cell pathways, CATS 2 in complement activation pathways, and CATS 3 in G-protein receptor signaling. CATS 1 had the lowest survival probability, while CATS 3 exhibited the highest [79]. Though the study lacked external validation, it represents an initial attempt to identify de novo transcriptional phenotypes in ARDS, offering potential for molecular classification in this complex syndrome.

While biological subphenotyping in critical care is rapidly evolving, no subphenotyping model has yet been integrated into clinical decision-making. For clinical integration, subphenotype-specific treatment efficacy must be prospectively evaluated in clinical trials. A significant hurdle remains the bedside identification of biological subphenotypes. Traditional LCA models for ARDS require 30–40 variables, making them impractical for real-time use. To address this, simpler models using just three to four variables—such as two protein biomarkers and clinical parameters—have been developed, achieving high accuracy with reduced complexity [80]. A proof-of-concept study demonstrated the feasibility of prospective bedside classification of ARDS subphenotypes in a small cohort of COVID-19 ARDS patients (N = 39), with the ongoing PHIND study (NCT04009330) aiming to validate these findings in a larger population. In addition, machine learning models trained on readily available clinical variables have been developed to classify ARDS subphenotypes with high accuracy, even when applied to electronic health record-derived data [81, 82]. These models preserved the biological and clinical characteristics of LCA-derived subphenotypes, demonstrating their potential for clinical implementation and personalized treatment strategies.

To further advance the identification of treatable traits in ARDS, future omics research should adopt integrated multi-omics approaches, combining genomics, proteomics, transcriptomics, metabolomics, lipidomics, and epigenomics. These integrated approaches will provide a more comprehensive understanding of disease mechanisms. Standardized protocols for sample collection, processing, and analysis will be essential to ensure reproducibility and facilitate cross-study comparisons. In addition, incorporating machine learning and artificial intelligence into these approaches will enhance patient stratification, enabling precise identification of biologically distinct subphenotypes and their responses to therapy. Implementing these methods will drive subphenotype-driven therapeutic strategies and pave the way for precision medicine in ARDS.

### Multi-omics approaches in ARDS

**Fig. 1:**
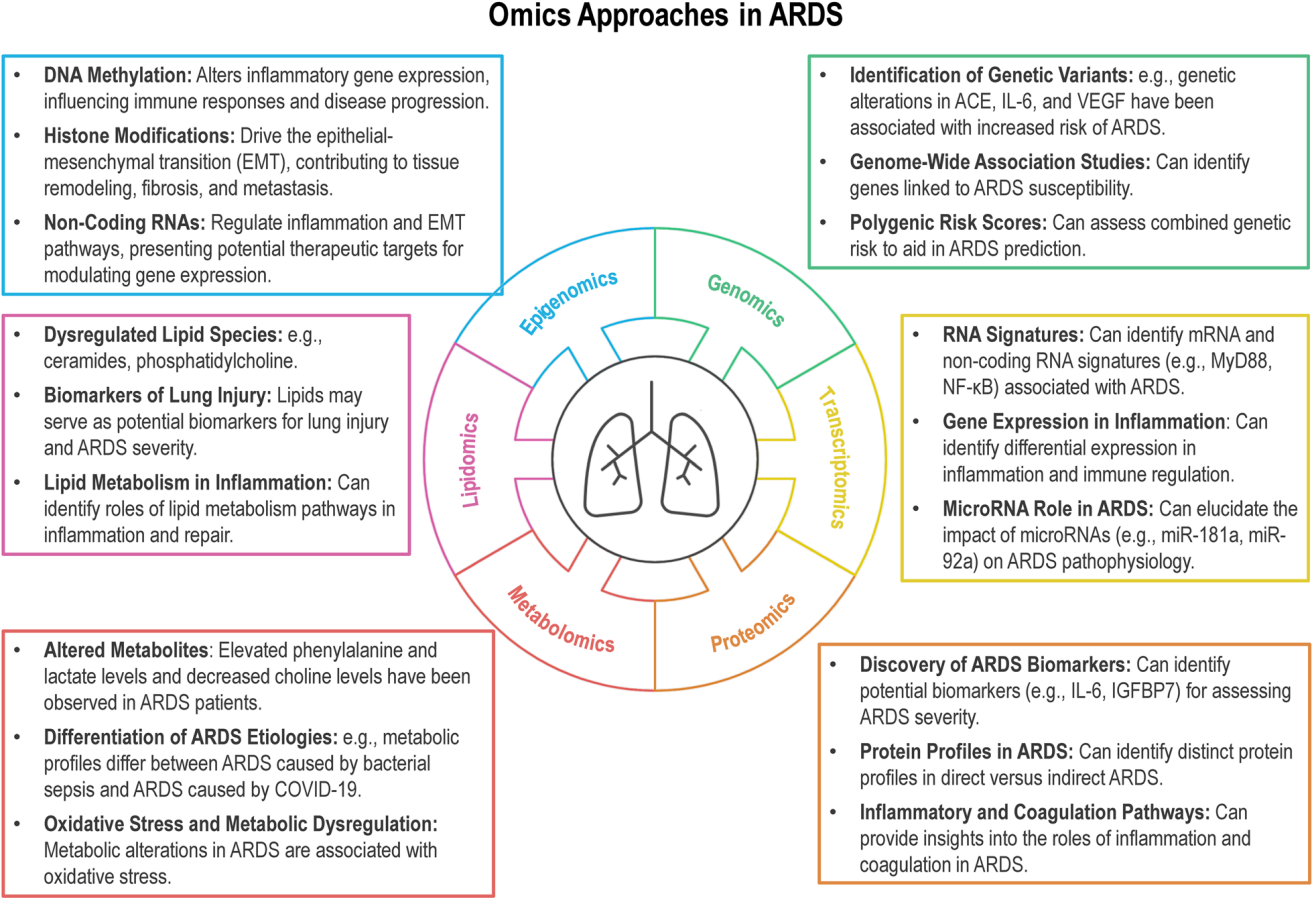
Omics ICMexp

The multi-omics approach integrates various biological data sets, such as the genome, transcriptome, epigenome, proteome, and lipidome, to better understand the pathophysiology of ARDS and identify associated biomarkers [83] (Fig. 1). Although multi-omics applications in ARDS are still in early stages, several studies have provided valuable insights [84]. These approaches offer a system-level understanding of ARDS but face challenges in data interpretation, clinical translation, and cost, which need to be addressed for broader clinical application. This section of the manuscript discusses the findings of studies that have explored the application of multi-omics approaches in ARDS.

Overmyer et al. conducted the first multi-omics analysis involving 102 COVID-19 patients and 26 non-COVID-19 patients to identify biomarkers related to COVID-19 severity [85]. They measured proteins, metabolites, lipids, and transcripts from blood samples using mass spectrometry and RNA sequencing. The study identified 219 biomolecules linked to COVID-19 susceptibility and severity, revealing disruptions in pathways, such as neutrophil degranulation, blood clotting, lipid transport, endothelial damage, and the acute phase response.

Liao et al. conducted a multi-omics study on 568 ARDS patients, analyzing data from GWAS, RNA sequencing, DNA methylation, and proteomics from urine samples [86]. Nine genes—NUP214, TNPO1, HNRNPA1, HDAC1, FOSB, GATAD2A, DDX17, CREBBP, and PHF20—were identified as potential predictors of ARDS mortality, with an area under the curve (AUC) of 0.83. These genes highlighted the role of IL-6, TP53, HDAC1, and TGF-β signaling pathways as key regulators of ARDS mortality. In addition, it was found that measuring angiopoietin 2 (Ang2) levels on day 7 could predict ARDS mortality, with an AUC of 0.70. The findings of Liao et al. suggest that the 9-gene panel and angiopoietin-2 levels could be developed into predictive biomarkers, guiding early clinical interventions to improve ARDS patient management.

In another study conducted by Batra et al., plasma-based metabolomics, lipidomics, and proteomics profiles were compared between ARDS caused by COVID-19 and bacterial sepsis [87]. The analysis revealed significant differences, including disrupted PI3K–AKT signaling, sphingosine metabolism, arginine metabolism, and MAPK signaling in COVID-19-induced ARDS. The study also highlighted the JAK–STAT signaling pathway as a potential therapeutic target and confirmed the role of specific molecules in developing acute kidney injury (AKI) and thrombosis.

Batra et al. compared urine-based metabolomic and proteomic profiles between 42 COVID-19 ARDS patients and 17 bacterial sepsis ARDS patients [88]. A total of 220 molecules—150 metabolites and 70 proteins—differed significantly between the two groups. The analysis suggested that extracellular matrix molecules, inflammation, and mitochondrial dysfunction are key contributors to ARDS pathogenesis. It was found that specific molecular changes correlated with clinical features, including the PaO2/FiO2 ratio, AKI, platelet count, and mortality in the COVID-19 ARDS group. In contrast, no such associations were found in the bacterial sepsis ARDS group. This suggests a proteomic signature associated with mortality in COVID-19 ARDS patients, highlighting potential predictive biomarkers.

Lin et al. analyzed 130 ARDS patients in a multicenter study, 33 non-ARDS ill controls, and 33 healthy individuals using 4D data-independent acquisition (4D-DIA) proteomics and global metabolomics [84]. They identified 36 proteins as potential ARDS biomarkers, with eight showing consistent significance across the discovery and validation groups. Dysregulation in oxidative phosphorylation and glycolysis pathways was also noted in ARDS patients, with predictive models based on these proteins performing better than traditional clinical models.

A phenome-wide Mendelian randomization study conducted by Cao et al. aimed to identify genetic causal factors of ARDS was conducted and a predictive model for early risk assessment was developed [89]. The study analyzed summary statistics from an ARDS GWAS involving 1,250 cases and 1,583 controls of European ancestry, assessing 33,150 traits for potential causal relationships with ARDS development. A total of 1,736 traits were identified, including 1,223 blood RNA markers, 159 plasma proteins, and 354 non-gene phenotypes across various biological categories. These findings were incorporated into a user-friendly platform, CARDS (Causal Traits for ARDS). To validate candidate biomarkers, transcriptomic data from human blood and lung tissues, along with a preclinical mouse model, were analyzed. Four genes (TMEM176B, SLC2A5, CDC45, and VSIG8) demonstrated differential and dynamic expression in ARDS patients and mouse lung tissues. A predictive model integrating these genes with five immune cell proportions significantly improved ARDS risk prediction (AUC = 0.791) compared to a traditional clinical model (AUC = 0.725). Finally, a nomogram was developed to facilitate clinical application, providing a promising tool for early ARDS risk stratification and intervention.

The reviewed studies collectively highlight several key molecular mechanisms underlying ARDS, emphasizing inflammation, coagulation dysregulation, metabolic dysfunction, biomarker discovery, and potential therapeutic targets. A common theme across multiple studies is the critical role of inflammatory pathways, particularly IL-6, TNF, TGF-β, and JAK–STAT signaling, in ARDS progression. These pathways contribute to the heightened inflammatory response seen in both COVID-19 and bacterial sepsis-induced ARDS, with prolonged immune activation distinguishing COVID-19-related cases. In addition, coagulation abnormalities and endothelial dysfunction are consistently reported, with studies identifying platelet activation, thrombosis, and extracellular matrix alterations as key contributors to ARDS severity. Metabolic and mitochondrial dysfunction also emerge as central features, with alterations in glycolysis, lipid transport, and arginine metabolism linked to disease progression. Several studies propose biomarkers for ARDS prognosis, including a 9-gene mortality signature (Liao et al.) [86], a proteomic mortality signature (Batra et al.) [88], and a 36-protein panel (Lin et al.) [84], all of which offer potential for early risk stratification. These insights further support therapeutic exploration, with JAK–STAT inhibitors (Batra et al.) [88], IL-6 and HDAC inhibitors (Liao et al.) [86], and metabolic modulators targeting glycolysis and sphingosine metabolism (Lin et al.) [84] emerging as promising avenues. Taken together, these findings underscore the complexity of ARDS pathophysiology and reinforce the potential of multi-omics approaches in identifying predictive biomarkers and targeted therapies to improve patient outcomes.

### Unresolved challenges and future directions in ARDS omics research

Despite significant progress, challenges persist across all omics fields in ARDS research—genomics, transcriptomics, proteomics, metabolomics, lipidomics, and epigenomics—hindering clinical translation. A major difficulty is the extreme heterogeneity of ARDS, arising from diverse etiologies and pathophysiologic triggers, which complicates the identification of universal biomarkers or targets. No single gene or molecular signature has been definitively linked to ARDS susceptibility or outcomes, reflecting the syndrome’s multifactorial nature. Although genome-wide studies have uncovered several risk variants, their effects tend to be modest and often population-specific, highlighting issues, such as limited cohort diversity and reproducibility. Similarly, transcriptomic and proteomic profiles vary widely, with results influenced by the source and timing of samples. Blood-based assays, in particular, may fail to capture lung-specific injury processes, especially since direct lung tissue or bronchoalveolar lavage (BAL) sampling is rarely feasible in ARDS. Epigenomic modifications add further complexity, as environmental factors such as mechanical ventilation and infection can induce epigenetic changes that influence gene expression without altering the DNA sequence. However, the contribution of these changes to ARDS risk and recovery remains poorly understood.

Omic studies also face significant data analysis challenges. Small sample sizes and patient heterogeneity make it difficult to discern which signals are truly related to the disease and which may be random or unrelated. Furthermore, practical and technical barriers—such as the need for specialized equipment, expertise, and high costs—limit the widespread adoption of omics analyses, creating a gap between research findings and bedside applications.

Looking ahead, efforts are underway to address these challenges and leverage omics for precision medicine in ARDS. Large multi-center collaborations and standardized biobanking of specimens are being developed to increase sample sizes, reduce inter-study variability, and facilitate the robust validation of findings. Advancements in bioinformatics are focused on integrative multi-omics analysis, aiming to synthesize genomic, transcriptomic, proteomic, metabolomic, and lipidomic data into coherent biological signatures while tackling methodological challenges, such as data harmonization and missing information. Incorporating longitudinal sampling is also a priority, as omics profiles over time may reveal dynamic changes in gene expression and metabolism that static snapshots miss. Importantly, researchers are emphasizing the functional validation of omics-derived biomarkers to improve interpretability. For example, determining whether a circulating metabolite identified in ARDS studies actively drives pathology or merely reflects tissue damage is crucial.

The ultimate goal is to translate omics insights into targeted therapies, and promising initiatives are beginning to bridge this gap. One such example is the ongoing PANTHER trial (Precision medicine Adaptive Network Trial in Hypoxaemic Respiratory Failure), an adaptive platform randomized clinical trial that stratifies ARDS patients into biological subphenotypes (e.g., hyperinflammatory vs. hypoinflammatory) based on biomarker profiles and then matches each subgroup with tailored interventions [90]. This precision-medicine approach will test whether treating patients according to an omics-informed phenotype improves outcomes, exemplifying how future ARDS trials can integrate biomarker stratification. These efforts, along with the continued expansion of omics consortia and rigorous validation in diverse populations, are expected to accelerate the development of clinically actionable diagnostics and personalized therapies for ARDS.

## Conclusions

Genomics, transcriptomics, proteomics, metabolomics, lipidomics, epigenomics, and multi-omics approaches are profoundly advancing our understanding of ARDS by offering detailed insights into the molecular and cellular mechanisms that drive this complex condition. As we move forward, refining and integrating these methodologies will be pivotal in improving patient stratification and treatment outcomes. There is an urgent need to develop personalized therapeutic strategies based on specific molecular profiles, which should enable targeted interventions tailored to the unique pathophysiology of each ARDS subtype.

Furthermore, advancements in machine learning and artificial intelligence hold great promise for enhancing the predictive accuracy of multi-omics data, ultimately leading to more reliable and clinically relevant models. Expanding these studies to encompass a broader spectrum of ARDS etiologies beyond COVID-19 will provide a more comprehensive understanding of the disease and its diverse clinical presentations.

The establishment of standardized protocols for sample collection, data processing, and analysis is essential to ensuring the reproducibility and reliability of findings. Such protocols will also facilitate collaboration and data sharing among research centers, fostering a more unified approach to ARDS research.

Performing bedside omics analyses is fraught with limitations, such as reliance on complex equipment, intricate sample preparation requirements, lengthy analysis times, high costs, and the need for specialized expertise in data interpretation. These challenges, which hinder both access to these technologies and reproducibility in research, are shared across multiple omics fields, including genomics, transcriptomics, and metabolomics. Addressing these systemic barriers is essential for integrating omics technologies into routine clinical practice and enhancing their utility for bedside diagnostics.

Ultimately, the integration of omics data with real-time clinical information will pave the way for dynamic, adaptive treatment strategies. These strategies have the potential to significantly improve outcomes for ARDS patients, making precision medicine a reality in this critical area of respiratory care.

## Supplementary Information

Supplementary Material 1.

## Data Availability

The data supporting the findings of this study are available within the article.

## Abbreviations

AKI: Acute kidney injury
AUC: Area under the curve
ARDS: Acute respiratory distress syndrome
BALF: Bronchoalveolar lavage fluid
BORCS5: BLOC-1-related complex subunit 5
CpG: Cytosine–phosphate–guanine
COVID-19: Coronavirus disease 2019
DUSP16: Dual specificity phosphatase 16
EMT: Epithelial–mesenchymal transition
EVs: Extracellular vesicles
FFR: Fps/Fes-related
FLT1: FMS-related receptor tyrosine kinase 1
GC–MS: Gas chromatography–mass spectrometry
GWAS: Genome-wide association studies
IFNAR: Interferon alpha/beta receptor
JAK–STAT: Janus kinase–signal transducer and activator of transcription
LC–MS/MS: Liquid chromatography–tandem mass spectrometry
lncRNA: Long non-coding RNA
LNPs: Lipid nanoparticles
MALDI: Matrix-assisted laser desorption/ionization
MAPK: Mitogen-activated protein kinase
mRNA: Messenger ribonucleic acid
miRNA: MicroRNA
MSCs: Mesenchymal stromal cells
NMR: Nuclear magnetic resonance
PD-1: Programmed death-1
PD-L1: Programmed cell death ligand 1
PPFIA1: Interacting protein 1
PRS: Polygenic risk scores
PTPRF: Protein tyrosine phosphatase receptor Type F polypeptide
RP-LC: Reversed-phase liquid chromatography
SELPL: Selectin P ligand
SNPs: Single-nucleotide polymorphisms
SpO₂/FiO₂: Peripheral oxygen saturation to fraction of inspired oxygen
TLR: Toll-like receptor
UHPLC–MS/MS: Ultrahigh-performance liquid chromatography–tandem mass spectrometry
WES: Whole-exome sequencing
WGS: Whole-genome sequencing
2D-GE: Two-dimensional gel electrophoresis
