# Supplementary material for: Advancing omics technologies in acute respiratory distress syndrome: paving the way for personalized medicine
Source: Intensive Care Med Exp. 2025 Jun 13;13:61. doi: 10.1186/s40635-025-00766-4 (PMC12165931; doi:10.1186/s40635-025-00766-4)
Supplement: Supplementary file 1 — Supplementary Material 1. [file 40635_2025_766_MOESM1_ESM.docx]

Supplementary Tables **Supplementary Table 1. Candidate gene studies in ARDS patients**

| **Author** | **Gene Variant** | **ARDS Susceptibility** | **ARDS Outcomes** |
| --- | --- | --- | --- |
| Marshall et al. (2002) [1] | *ACE* D-allele and IL-6 C-allele | *ACE* D-allele and DD-genotype increased susceptibility | *ACE* D-allele and DD-genotype increased mortality IL-6 C-allele and CC-genotype improved survival |
| Ye et al. (2005) [2] | *PBEF* T-1001G and C-1543T | T-1001G and C-1543T were associated with increased ARDS risk | Not reported |
| Marzec et al. (2007) [3] | NRF2 | NRF2 -617 A was associated with increased ARDS risk | Not reported |
| Sapru et al. (2009) [4] | PAI-1 | Not reported | 4G allele was associated with fewer ventilator-free days and increased mortality |
| Meyer et al. (2013) [5] | *IL1RN* rs315952 | IL1RN rs315952 was associated with reduced ARDS risk | Not reported |
| Tejera et al. (2014) [6] | *PI3* rs2664581 | C allele was associated with increased ARDS risk | Not reported |
| Wei et al. (2015) [7] | *LRRC16A* rs7766874 | Increased | Not reported |
| Hinz et al. (2017) [8] | *FER* rs4957796 | Not reported | Higher 90-day mortality risk among T homozygotes than among C-allele carriers |
| Wei et al. (2017) [9] | *LRRC16A* rs9358856G>A |  | Favorable survival within 28 days |
| Dötsch et al. (2017) [10] | *PHD2* rs516651 | Not reported | TT-genotype increased mortality |
| Jabaudon et al. (2018) [11] | *AGER* rs2070600 | Increased | Increased mortality |
| Reilly et al. (2018) [12] | *ANG2* rs2442608C | Increased | Not reported |
| Hernandez-Pacheco et al. (2018) [13] | *FLT1* rs9513106 | C allele was associated with increased ARDS risk | Not reported |
| Morrell et al. (2018) [14] | *MAP3K1*  rs832582 | Not reported | Decreased ventilator-free days, increased inflammatory burden, increased mortality (not significant) |
| Rogers et al. (2018) [15] | *MUC5B* rs35705950 | Increased | Not reported |
| Feng et al. (2019) [16] | HBD-1 rs1800972 | Increased | Decreased survival |
| Xie et al. (2019) [17] | IL-17 rs763780, rs2275913, and rs8193036.  NF-κB1 gene rs3774934 (G>A) | IL-17 rs763780 and NF-κB1 rs3774934 SNPs had no impact on ARDS risk and prognosis.  AA-homozygosity and GA- heterozygosity for IL-17 rs2275913 reduced ARDS risk, while TT- homozygote and CT-heterozygote genotypes of IL-17 rs2275913 increased ARDS risk | AA-homozygous and GA- heterozygous genotypes of IL-17 rs2275913 reduced ARDS mortality, while TT- homozygote and CT- heterozygote genotypes of IL-17 rs2275913 increased ARDS mortality |
| Ding et al. (2019) [18] | TNF-α rs1800629, IL-6 rs1800796, and MyD88 rs7744 | The *TNF-α* rs1800629 locus A allele and the *IL-6* rs1800796 locus G allele were risk factors for ARDS. The G allele at *MyD88* rs7744 locus was a protective factor against ARDS | Homozygotes for TNF-α rs1800629 and IL-6 rs1800796 loci had lower 60-day survival rates, while MyD88 rs7744 locus homozygotes had a higher 60-day survival rate |
| Ahasic et al. (2014) [19] | *ADIPOQ*, *ADIPOR1*, *ADIPOR2* |  | Homozygotes for the minor allele of rs2082940 (*ADIPOQ*) had increased mortality |
| Jalkanen et al. (2023) [21] | *IFNAR* rs9984273 | Not reported | Minor allele improved survival; major allele increased mortality with glucocorticoids |
| Lynn et al. (2023) [22] | *NAMPT* G-1535A, A-1001 C, and C-948A | G-1535A and C-948A SNPs were significantly associated with ARDS | Not reported |

**Supplementary Table 2**. Genome-wide association studies in ARDS patients

| **Author** | **Model** | **Population** | **Mechanism of ARDS** | **Genes** |
| --- | --- | --- | --- | --- |
| Tejera et al. (2012) [23] | Multistage genetic association study | Caucasian | Pulmonary or extrapulmonary injury | *POPDC3* (pulmonary causes), *FAAH* (extrapulmonary causes) |
| Christie et al. (2012) [24] | Genome-wide association study | European American | Trauma | *PPFIA1* |
| Bime et al. (2018) [25] | Genome-wide association study | African American | Sepsis, ventilator-induced lung injury (VILI) | *SELPLG* |
| Guillen-Guio et al. (2020) [26] | Genome-wide association study | European | Sepsis | *FLT1* |
| Du et al. (2021) [27] | Meta-analysis of ARDS genome-wide association studies | Europeans and African Americans | Sepsis | *BORCS5* and *DUSP16* |
| Mousa et al. (2021) [28] | Trans-ancestry, genome-wide association study | Emiratis | COVID-19 | *VWA8*, *PDE8B*, *CTSC*, *THSD7B*, *STK39*, *FBXO34*, *RPL6P27*, *METTL21C* |
| Cao et al. (2024) [29] | Phenome-wide Mendelian randomization analysis of a genome-wide association study | Europeans | Sepsis | *TMEM176B*, *SLC2A5*, *CDC45*, and *VSIG8* |

**Supplementary Table 3.** Summary of miRNA studies in ARDS patients

| **Author** | **Micro-RNA** | **Model** | **Effect** |
| --- | --- | --- | --- |
| Zhang et al. (2021)  [30] | miR-584, miR-146a | Serum samples from ARDS patients | Both miRNAs were downregulated in ARDS patients |
| Goodwin et al. (2020)  [31] | miR-887-3p | Plasma from sepsis patients | Elevated in ARDS patients; increased the expression of ARDS-related genes (*CCL5*, *CXCL10*, *VCAM1*, *CX3CL1*, *CASP1*, *IFNB*, *IL1B*, and *TLR2I*), altered endothelial gene expression, increased leukocyte migration |
| Rahmel et al. (2018)  [32] | miR-122 | Serum from ARDS patients | Elevated in non-survivors; correlated with liver function markers |
| Zhu et al. (2017)  [33] | miR-181a, miR-92a, miR-424 | Whole blood from ARDS patients | miR-181a and miR-92a: risk biomarkers; miR-424: protective biomarker |
| Lu et al. (2017)  [34] | miR-1260b, miR-762, miR-22-3p, miR-23b, miR-23a, miR-550a*, miR-324-5p, miR-484, miR-331-3p, miR-140-3p, miR-342-3p, miR-150 | Severe acute pancreatitis patients with ALI | Differential expression in severe acute pancreatitis with ALI |
| Long et al. (2024)  [35] | miR-141-3p | Serum samples from ARDS patients (n=137) and healthy controls (n=69) | Downregulated in ARDS patients; associated with higher Murray and APACHEII scores and occurrence of PF |
| Hu et al. (2023)  [36] | miR-574-5p | Human samples; LPS-treated WI-38 cells | circ_0001498 promotes ALI progression by downregulating miR-574-5p and upregulating SOX6 |
| Curcio et al. (2023)  [37] | miR-17-5p, miR-146a-5p, miR-223-3p | Samples from COVID-19-related ARDS survivors | Downregulation of miR-17-5p, miR-146a-5p, and miR-223-3p correlated with fibrosis in COVID-19-related ARDS survivors |
| Najafipour et al. (2022)  [38] |  | Blood and BALF samples from COVID-19 ARDS patients | In BALF samples, miR-282, miR-15-5p, miR-4485-3p, miR-483-3p, miR-6891-5p, miR-200c, miR-4463, miR-483-5p, and miR-98-5p were upregulation, while miR-15a-5p, miR-548c-5p, miR-548d-3p, miR-365a-3p, miR-3939, miR-514-b-5p, miR-513a-3p, miR-513a-5p, miR-664a-3p, and miR-766-3p were downregulated. Conversely, in blood samples, the most upregulated miRNAs included miR-15b-5p, miR-18a-3p, miR-486-3p, miR-486-5p, miR-146a-5p, miR-16-2-3p, miR-6501-5p, miR-365-3p, miR-618, and miR-623, while miR-21-5p, miR-142a-3p, miR-181-a, miR-31-5p, miR-99-5p, miR-342-5p, miR-183-5p, miR-627-5p, and miR-144-3p were significantly downregulated |
| Xu et al. (2020)  [39] | miR-92a | Serum and cells samples in sepsis-induced ARDS patients | Elevated levels of miR-92a in sepsis-induced ARDS |
| García-Hidalgo et al. (2022)  [40] |  | Serum samples in COVID-19-induced ARDS | Elevated miR-17-5p, miR-27a-3p, miR-126-3p, miR-146a-5p and miR-495-3p were associated with compromised lung diffusion capacity in ARDS patients. Elevated miR-9-5p, miR-21-5p, miR-24-3p and miR-221-3p were associated with concerning radiologic features in ARDS patients |
| Zheng et al. (2018)  [41] | miR-221 and miR-27b | Blood samples of pulmonary and extrapulmonary ARDS patients | Plasma miR-221 and miR-27b levels were lower in extrapulmonary ARDS. Low levels of both miRNAs were associated with survival |
| Parzibut et al. (2021)  [42] | miR-130a-3p, miR-221-3p, miR-24-3p, miR-98-3p, Let-7d-3p, miR-1273a, and miR-193a-5p | Blood samples of ARDS patients | Elevated levels of miR-130a-3p, miR-221-3p, miR-24-3p, miR-98-3p, Let-7d-3p, miR-1273a, and miR-193a-5p were observed in ARDS patients |
| Wang et al. (2020)  [43] | miR-155 | Blood samples of sepsis-induced ARDS patients | Elevated levels of miR-155 correlated positively with tumor necrosis factor (TNF)-α and interleukin (IL)-1β levels and negatively with PaO2/FiO2 |
| Huang et al. (2022)  [44] | miR-155-5p | Blood samples of ARDS patients | Elevated levels of miR-155-5p, miR-155-5p alleviate lung injury in cell module by inhibiting the expression of interleukin receptors |
| Almuntashiri et al. (2022)  [45] | miR-223 | Blood samples of ARDS patients | Elevated levels of circulating microvesicle-containing miR-223 were associated with ARDS risk and 30-day mortality |
| Garcia-Giralt et al. (2022)  [46] | miR-369-3p | Serum samples in COVID-19-induced ARDS | Elevated levels of circulating miR-369-3p were found in ARDS patients who required mechanical ventilation |
| Meidert et al. (2021)  [47] |  | Serum samples in COVID-19-induced ARDS | 20 miRNAs (15 decreased levels) in COVID-19 ARDS patients in comparison to those with COVID-19 pneumonia |
| Li et al. (2020)  [48] | miR-125a and miR-125b | Blood samples in sepsis-induced ARDS | miR-125a was elevated in sepsis-induced ARDS patients |
| Zhang et al. (2024)  [49] | miR-486-3p | Serum samples of ARDS patients with or without pulmonary fibrosis | miR-486-3p was significantly different in both ARDS and pulmonary fibrosis patients |
| Wang et al. (2020)  [50] | miR-103 and miR-107 | Plasma samples of sepsis-induced ARDS | Reduced miR-103 and miR-107 levels were corelated sepsis-induced ARDS risk and mortality. |
| Chen et al. (2020)  [51] | miR-146b | Plasma samples of sepsis-induced ARDS | Reduced miR-146b levels in sepsis-induced ARDS |
| Scheller et al. (2019)  [52] | miR-17-5p | Bronchoalveolar lavage fluid in influenza A virus-induced ARDS | Elevated levels of miR-17-5p |
| Zhu et al. (2016)  [53] | miR-628.3p, miR-922, miR-505, miR-130b, and miR-624 | Blood samples of ICU ARDS patients | Elevated miR-628.3p, miR-922, miR-505, miR-130b, and miR-624 levels were associated with ARDS mortality |

**References**

1. Marshall, R.P.; Webb, S.; Hill, M.R.; Humphries, S.E.; Laurent, G.J. Genetic polymorphisms associated with susceptibility and outcome in ARDS. *Chest* **2002**, *121*, 68s-69s, doi:10.1378/chest.121.3_suppl.68s.

2. Ye, S.Q.; Simon, B.A.; Maloney, J.P.; Zambelli-Weiner, A.; Gao, L.; Grant, A.; Easley, R.B.; McVerry, B.J.; Tuder, R.M.; Standiford, T.; et al. Pre-B-cell colony-enhancing factor as a potential novel biomarker in acute lung injury. *American journal of respiratory and critical care medicine* **2005**, *171*, 361-370, doi:10.1164/rccm.200404-563OC.

3. Marzec, J.M.; Christie, J.D.; Reddy, S.P.; Jedlicka, A.E.; Vuong, H.; Lanken, P.N.; Aplenc, R.; Yamamoto, T.; Yamamoto, M.; Cho, H.Y.; et al. Functional polymorphisms in the transcription factor NRF2 in humans increase the risk of acute lung injury. *FASEB journal : official publication of the Federation of American Societies for Experimental Biology* **2007**, *21*, 2237-2246, doi:10.1096/fj.06-7759com.

4. Sapru, A.; Hansen, H.; Ajayi, T.; Brown, R.; Garcia, O.; Zhuo, H.; Wiemels, J.; Matthay, M.A.; Wiener-Kronish, J. 4G/5G polymorphism of plasminogen activator inhibitor-1 gene is associated with mortality in intensive care unit patients with severe pneumonia. *Anesthesiology* **2009**, *110*, 1086-1091, doi:10.1097/ALN.0b013e3181a1081d.

5. Meyer, N.J.; Feng, R.; Li, M.; Zhao, Y.; Sheu, C.C.; Tejera, P.; Gallop, R.; Bellamy, S.; Rushefski, M.; Lanken, P.N.; et al. IL1RN coding variant is associated with lower risk of acute respiratory distress syndrome and increased plasma IL-1 receptor antagonist. *American journal of respiratory and critical care medicine* **2013**, *187*, 950-959, doi:10.1164/rccm.201208-1501OC.

6. Tejera, P.; O'Mahony, D.S.; Owen, C.A.; Wei, Y.; Wang, Z.; Gupta, K.; Su, L.; Villar, J.; Wurfel, M.; Christiani, D.C. Functional characterization of polymorphisms in the peptidase inhibitor 3 (elafin) gene and validation of their contribution to risk of acute respiratory distress syndrome. *American journal of respiratory cell and molecular biology* **2014**, *51*, 262-272, doi:10.1165/rcmb.2013-0238OC.

7. Wei, Y.; Wang, Z.; Su, L.; Chen, F.; Tejera, P.; Bajwa, E.K.; Wurfel, M.M.; Lin, X.; Christiani, D.C. Platelet count mediates the contribution of a genetic variant in LRRC16A to ARDS risk. *Chest* **2015**, *147*, 607-617, doi:10.1378/chest.14-1246.

8. Hinz, J.; Büttner, B.; Kriesel, F.; Steinau, M.; Frederik Popov, A.; Ghadimi, M.; Beissbarth, T.; Tzvetkov, M.; Bergmann, I.; Mansur, A. The FER rs4957796 TT genotype is associated with unfavorable 90-day survival in Caucasian patients with severe ARDS due to pneumonia. *Scientific Reports* **2017**, *7*, 9887, doi:10.1038/s41598-017-08540-7.

9. Wei, Y.; Tejera, P.; Wang, Z.; Zhang, R.; Chen, F.; Su, L.; Lin, X.; Bajwa, E.K.; Thompson, B.T.; Christiani, D.C. A Missense Genetic Variant in LRRC16A/CARMIL1 Improves Acute Respiratory Distress Syndrome Survival by Attenuating Platelet Count Decline. *American journal of respiratory and critical care medicine* **2017**, *195*, 1353-1361, doi:10.1164/rccm.201605-0946OC.

10. Dötsch, A.; Eisele, L.; Rabeling, M.; Rump, K.; Walstein, K.; Bick, A.; Cox, L.; Engler, A.; Bachmann, H.S.; Jöckel, K.H.; et al. Hypoxia Inducible Factor-2 Alpha and Prolinhydroxylase 2 Polymorphisms in Patients with Acute Respiratory Distress Syndrome (ARDS). *International journal of molecular sciences* **2017**, *18*, doi:10.3390/ijms18061266.

11. Jabaudon, M.; Berthelin, P.; Pranal, T.; Roszyk, L.; Godet, T.; Faure, J.S.; Chabanne, R.; Eisenmann, N.; Lautrette, A.; Belville, C.; et al. Receptor for advanced glycation end-products and ARDS prediction: a multicentre observational study. *Sci Rep* **2018**, *8*, 2603, doi:10.1038/s41598-018-20994-x.

12. Reilly, J.P.; Wang, F.; Jones, T.K.; Palakshappa, J.A.; Anderson, B.J.; Shashaty, M.G.S.; Dunn, T.G.; Johansson, E.D.; Riley, T.R.; Lim, B.; et al. Plasma angiopoietin-2 as a potential causal marker in sepsis-associated ARDS development: evidence from Mendelian randomization and mediation analysis. *Intensive care medicine* **2018**, *44*, 1849-1858, doi:10.1007/s00134-018-5328-0.

13. Hernandez-Pacheco, N.; Guillen-Guio, B.; Acosta-Herrera, M.; Pino-Yanes, M.; Corrales, A.; Ambrós, A.; Nogales, L.; Muriel, A.; González-Higueras, E.; Diaz-Dominguez, F.J.; et al. A vascular endothelial growth factor receptor gene variant is associated with susceptibility to acute respiratory distress syndrome. *Intensive care medicine experimental* **2018**, *6*, 16, doi:10.1186/s40635-018-0181-6.

14. Morrell, E.D.; O'Mahony, D.S.; Glavan, B.J.; Harju-Baker, S.; Nguyen, C.; Gunderson, S.; Abrahamson, A.; Radella, F., 2nd; Rona, G.; Black, R.A.; et al. Genetic Variation in MAP3K1 Associates with Ventilator-Free Days in Acute Respiratory Distress Syndrome. *American journal of respiratory cell and molecular biology* **2018**, *58*, 117-125, doi:10.1165/rcmb.2017-0030OC.

15. Rogers, A.J.; Solus, J.F.; Hunninghake, G.M.; Baron, R.M.; Meyer, N.J.; Janz, D.R.; Schwartz, D.A.; May, A.K.; Lawson, W.E.; Blackwell, T.S.; et al. MUC5B Promoter Polymorphism and Development of Acute Respiratory Distress Syndrome. *American journal of respiratory and critical care medicine* **2018**, *198*, 1342-1345, doi:10.1164/rccm.201801-0140LE.

16. Feng, Q.; Liu, N.; Song, S.; Ma, Y. Relationship between β-defensin-1 gene polymorphism and susceptibility and prognosis of acute respiratory distress syndrome. *Medicine* **2019**, *98*, e14131, doi:10.1097/md.0000000000014131.

17. Xie, M.; Cheng, B.; Ding, Y.; Wang, C.; Chen, J. Correlations of IL-17 and NF-κB gene polymorphisms with susceptibility and prognosis in acute respiratory distress syndrome in a chinese population. *Bioscience reports* **2019**, *39*, doi:10.1042/bsr20181987.

18. Ding, Y.; Feng, Q.; Chen, J.; Song, J. TLR4/NF-κB signaling pathway gene single nucleotide polymorphisms alter gene expression levels and affect ARDS occurrence and prognosis outcomes. *Medicine* **2019**, *98*, e16029, doi:10.1097/md.0000000000016029.

19. Ahasic, A.M.; Zhao, Y.; Su, L.; Sheu, C.C.; Thompson, B.T.; Christiani, D.C. Adiponectin gene polymorphisms and acute respiratory distress syndrome susceptibility and mortality. *PloS one* **2014**, *9*, e89170, doi:10.1371/journal.pone.0089170.

20. Yao, R.; Chen, T.; Xue, F. The association of IL-10-1082G/A gene polymorphism with the risk of acute lung injury/respiratory distress syndrome (ALI/RDS): A meta-analysis. *Heart & lung : the journal of critical care* **2023**, *58*, 158-165, doi:10.1016/j.hrtlng.2022.11.019.

21. Jalkanen, J.; Khan, S.; Elima, K.; Huttunen, T.; Wang, N.; Hollmén, M.; Elo, L.L.; Jalkanen, S. Polymorphism in interferon alpha/beta receptor contributes to glucocorticoid response and outcome of ARDS and COVID-19. *Crit Care* **2023**, *27*, 112, doi:10.1186/s13054-023-04388-8.

22. Lynn, H.; Sun, X.; Casanova, N.G.; Bime, C.; Reyes Hernon, V.; Lanham, C.; Oita, R.C.; Ramos, N.; Sun, B.; Coletta, D.K.; et al. Linkage of NAMPT promoter variants to eNAMPT secretion, plasma eNAMPT levels, and ARDS severity. *Therapeutic advances in respiratory disease* **2023**, *17*, 17534666231181262, doi:10.1177/17534666231181262.

23. Tejera, P.; Meyer, N.J.; Chen, F.; Feng, R.; Zhao, Y.; O'Mahony, D.S.; Li, L.; Sheu, C.C.; Zhai, R.; Wang, Z.; et al. Distinct and replicable genetic risk factors for acute respiratory distress syndrome of pulmonary or extrapulmonary origin. *Journal of medical genetics* **2012**, *49*, 671-680, doi:10.1136/jmedgenet-2012-100972.

24. Christie, J.D.; Wurfel, M.M.; Feng, R.; O'Keefe, G.E.; Bradfield, J.; Ware, L.B.; Christiani, D.C.; Calfee, C.S.; Cohen, M.J.; Matthay, M.; et al. Genome wide association identifies PPFIA1 as a candidate gene for acute lung injury risk following major trauma. *PloS one* **2012**, *7*, e28268, doi:10.1371/journal.pone.0028268.

25. Bime, C.; Pouladi, N.; Sammani, S.; Batai, K.; Casanova, N.; Zhou, T.; Kempf, C.L.; Sun, X.; Camp, S.M.; Wang, T.; et al. Genome-Wide Association Study in African Americans with Acute Respiratory Distress Syndrome Identifies the Selectin P Ligand Gene as a Risk Factor. *American journal of respiratory and critical care medicine* **2018**, *197*, 1421-1432, doi:10.1164/rccm.201705-0961OC.

26. Guillen-Guio, B.; Lorenzo-Salazar, J.M.; Ma, S.F.; Hou, P.C.; Hernandez-Beeftink, T.; Corrales, A.; García-Laorden, M.I.; Jou, J.; Espinosa, E.; Muriel, A.; et al. Sepsis-associated acute respiratory distress syndrome in individuals of European ancestry: a genome-wide association study. *The Lancet. Respiratory medicine* **2020**, *8*, 258-266, doi:10.1016/s2213-2600(19)30368-6.

27. Du, M.; Garcia, J.G.N.; Christie, J.D.; Xin, J.; Cai, G.; Meyer, N.J.; Zhu, Z.; Yuan, Q.; Zhang, Z.; Su, L.; et al. Integrative omics provide biological and clinical insights into acute respiratory distress syndrome. *Intensive care medicine* **2021**, *47*, 761-771, doi:10.1007/s00134-021-06410-5.

28. Mousa, M.; Vurivi, H.; Kannout, H.; Uddin, M.; Alkaabi, N.; Mahboub, B.; Tay, G.K.; Alsafar, H.S. Genome-wide association study of hospitalized COVID-19 patients in the United Arab Emirates. *EBioMedicine* **2021**, *74*, 103695, doi:10.1016/j.ebiom.2021.103695.

29. Cao, S.; Li, H.; Xin, J.; Jin, Z.; Zhang, Z.; Li, J.; Zhu, Y.; Su, L.; Huang, P.; Jiang, L.; et al. Identification of genetic profile and biomarkers involved in acute respiratory distress syndrome. *Intensive care medicine* **2024**, *50*, 46-55, doi:10.1007/s00134-023-07248-9.

30. Zhang, S.; Hong, Y.; Liu, H.; Wang, Q.; Xu, J.; Zhang, Y.; Zhao, X.; Yao, Y.; Zhou, K.; Ding, X. miR-584 and miR-146 are candidate biomarkers for acute respiratory distress syndrome. *Experimental and therapeutic medicine* **2021**, *21*, 445, doi:10.3892/etm.2021.9873.

31. Goodwin, A.J.; Li, P.; Halushka, P.V.; Cook, J.A.; Sumal, A.S.; Fan, H. Circulating miRNA 887 is differentially expressed in ARDS and modulates endothelial function. *American journal of physiology. Lung cellular and molecular physiology* **2020**, *318*, L1261-l1269, doi:10.1152/ajplung.00494.2019.

32. Rahmel, T.; Rump, K.; Adamzik, M.; Peters, J.; Frey, U.H. Increased circulating microRNA-122 is associated with mortality and acute liver injury in the acute respiratory distress syndrome. *BMC anesthesiology* **2018**, *18*, 75, doi:10.1186/s12871-018-0541-5.

33. Zhu, Z.; Liang, L.; Zhang, R.; Wei, Y.; Su, L.; Tejera, P.; Guo, Y.; Wang, Z.; Lu, Q.; Baccarelli, A.A.; et al. Whole blood microRNA markers are associated with acute respiratory distress syndrome. *Intensive Care Med Exp* **2017**, *5*, 38, doi:10.1186/s40635-017-0155-0.

34. Lu, X.G.; Kang, X.; Zhan, L.B.; Kang, L.M.; Fan, Z.W.; Bai, L.Z. Circulating miRNAs as biomarkers for severe acute pancreatitis associated with acute lung injury. *World journal of gastroenterology* **2017**, *23*, 7440-7449, doi:10.3748/wjg.v23.i41.7440.

35. Long, G.; Zhang, Q.; Yang, X.; Sun, H.; Ji, C. Diagnostic and Predictive Significance of Serum MiR-141-3p in Acute Respiratory Distress Syndrome Patients with Pulmonary Fibrosis. *Tohoku J Exp Med* **2024**, *262*, 157-162, doi:10.1620/tjem.2023.J093.

36. Hu, W.; Wang, Q.; Luo, Z.; Shi, Y.; Zhang, L.; Zhang, Z.; Liu, J.; Liu, K. Circ_0001498 contributes to lipopolysaccharide-induced lung cell apoptosis and inflammation in sepsis-related acute lung injury via upregulating SOX6 by interacting with miR-574-5p. *General physiology and biophysics* **2023**, *42*, 37-47, doi:10.4149/gpb_2022054.

37. Curcio, R.; Poli, G.; Fabi, C.; Sugoni, C.; Pasticci, M.B.; Ferranti, R.; Rossi, M.; Folletti, I.; Sanesi, L.; Santoni, E.; et al. Exosomal miR-17-5p, miR-146a-3p, and miR-223-3p Correlate with Radiologic Sequelae in Survivors of COVID-19-Related Acute Respiratory Distress Syndrome. *International journal of molecular sciences* **2023**, *24*, doi:10.3390/ijms241713037.

38. Najafipour, R.; Mohammadi, D.; Estaki, Z.; Zarabadi, K.; Jalilvand, M.; Moghbelinejad, S. Screening for differentially expressed microRNAs in BALF and blood samples of infected COVID-19 ARDS patients by small RNA deep sequencing. *Journal of clinical laboratory analysis* **2022**, *36*, e24672, doi:10.1002/jcla.24672.

39. Xu, F.; Yuan, J.; Tian, S.; Chen, Y.; Zhou, F. MicroRNA-92a serves as a risk factor in sepsis-induced ARDS and regulates apoptosis and cell migration in lipopolysaccharide-induced HPMEC and A549 cell injury. *Life sciences* **2020**, *256*, 117957, doi:10.1016/j.lfs.2020.117957.

40. García-Hidalgo, M.C.; González, J.; Benítez, I.D.; Carmona, P.; Santisteve, S.; Pérez-Pons, M.; Moncusí-Moix, A.; Gort-Paniello, C.; Rodríguez-Jara, F.; Molinero, M.; et al. Identification of circulating microRNA profiles associated with pulmonary function and radiologic features in survivors of SARS-CoV-2-induced ARDS. *Emerging microbes & infections* **2022**, *11*, 1537-1549, doi:10.1080/22221751.2022.2081615.

41. Zheng, Y.; Liu, S.Q.; Sun, Q.; Xie, J.F.; Xu, J.Y.; Li, Q.; Pan, C.; Liu, L.; Huang, Y.Z. Plasma microRNAs levels are different between pulmonary and extrapulmonary ARDS patients: a clinical observational study. *Annals of intensive care* **2018**, *8*, 23, doi:10.1186/s13613-018-0370-1.

42. Parzibut, G.; Henket, M.; Moermans, C.; Struman, I.; Louis, E.; Malaise, M.; Louis, R.; Misset, B.; Njock, M.S.; Guiot, J. A Blood Exosomal miRNA Signature in Acute Respiratory Distress Syndrome. *Frontiers in molecular biosciences* **2021**, *8*, 640042, doi:10.3389/fmolb.2021.640042.

43. Wang, Z.F.; Yang, Y.M.; Fan, H. Diagnostic value of miR-155 for acute lung injury/acute respiratory distress syndrome in patients with sepsis. *The Journal of international medical research* **2020**, *48*, 300060520943070, doi:10.1177/0300060520943070.

44. Huang, Z.; Huang, H.; Shen, M.; Li, C.; Liu, C.; Zhu, H.; Zhang, W. MicroRNA-155-5p modulates the progression of acute respiratory distress syndrome by targeting interleukin receptors. *Bioengineered* **2022**, *13*, 11732-11741, doi:10.1080/21655979.2022.2071020.

45. Almuntashiri, S.; Han, Y.; Youngblood, H.A.; Chase, A.; Zhu, Y.; Wang, X.; Linder, D.F.; Siddiqui, B.; Sikora, A.; Liu, Y.; et al. Identification of circulating microvesicle-encapsulated miR-223 as a potential novel biomarker for ARDS. *Physiological reports* **2022**, *10*, e15494, doi:10.14814/phy2.15494.

46. Garcia-Giralt, N.; Du, J.; Marin-Corral, J.; Bódalo-Torruella, M.; Blasco-Hernando, F.; Muñoz-Bermúdez, R.; Clarós, M.; Nonell, L.; Perera-Bel, J.; Fernandez-González, M.; et al. Circulating microRNA profiling is altered in the acute respiratory distress syndrome related to SARS-CoV-2 infection. *Sci Rep* **2022**, *12*, 6929, doi:10.1038/s41598-022-10738-3.

47. Meidert, A.S.; Hermann, S.; Brandes, F.; Kirchner, B.; Buschmann, D.; Billaud, J.N.; Klein, M.; Lindemann, A.; Aue, E.; Schelling, G.; et al. Extracellular Vesicle Associated miRNAs Regulate Signaling Pathways Involved in COVID-19 Pneumonia and the Progression to Severe Acute Respiratory Corona Virus-2 Syndrome. *Frontiers in immunology* **2021**, *12*, 784028, doi:10.3389/fimmu.2021.784028.

48. Li, S.; Zhao, D.; Cui, J.; Wang, L.; Ma, X.; Li, Y. Correlation of microRNA-125a/b with acute respiratory distress syndrome risk and prognosis in sepsis patients. *Journal of clinical laboratory analysis* **2020**, *34*, e23098, doi:10.1002/jcla.23098.

49. Zhang, S.; Rong, L.; Long, G.; Huang, F.; Zhang, Q.; Yang, X.; Sun, H.; Ji, C.; Ye, R.H. Clinical significance and potential mechanism of hsa_circ_0006892 in acute respiratory distress syndrome complicated with pulmonary fibrosis. *Molecular biology reports* **2024**, *51*, 1120, doi:10.1007/s11033-024-10047-0.

50. Wang, Q.; Feng, Q.; Zhang, Y.; Zhou, S.; Chen, H. Decreased microRNA 103 and microRNA 107 predict increased risks of acute respiratory distress syndrome and 28-day mortality in sepsis patients. *Medicine* **2020**, *99*, e20729, doi:10.1097/md.0000000000020729.

51. Chen, W.; Liu, L.; Yang, J.; Wang, Y. MicroRNA-146b correlates with decreased acute respiratory distress syndrome risk, reduced disease severity, and lower 28-day mortality in sepsis patients. *Journal of clinical laboratory analysis* **2020**, *34*, e23510, doi:10.1002/jcla.23510.

52. Scheller, N.; Herold, S.; Kellner, R.; Bertrams, W.; Jung, A.L.; Janga, H.; Greulich, T.; Schulte, L.N.; Vogelmeier, C.F.; Lohmeyer, J.; et al. Proviral MicroRNAs Detected in Extracellular Vesicles From Bronchoalveolar Lavage Fluid of Patients With Influenza Virus-Induced Acute Respiratory Distress Syndrome. *The Journal of infectious diseases* **2019**, *219*, 540-543, doi:10.1093/infdis/jiy554.

53. Zhu, Z.; Zhang, R.; Liang, L.; Su, L.; Lu, Q.; Baccarelli, A.A.; Bajwa, E.K.; Thompson, B.T.; Christiani, D.C. Whole blood microRNAs as a prognostic classifier for acute respiratory distress syndrome 28-day mortality. *Intensive care medicine* **2016**, *42*, 1824-1825, doi:10.1007/s00134-016-4462-9.
